# Association of Low Birth Weight Infants and Maternal Sociodemographic Status in Tuzla Canton during 1992–1995 War Period in Bosnia and Herzegovina

**DOI:** 10.1155/2010/789183

**Published:** 2011-03-28

**Authors:** Fahrija Skokić, Dubravka Bačaj, Amela Selimović, Evlijana Hasanović, Selma Muratović, Amir Halilbašić

**Affiliations:** Clinic for Children's Diseases, Tuzla University Clinical Centre, 75000 Tuzla, Bosnia and Herzegovina

## Abstract

*Objectives*. We examined association between incidence rate of low birth weight in liveborn infants and maternal sociodemographic status in Tuzla Canton during 1992–1995 war in Bosnia and Herzegovina. *Methods*. The present study covers a 22-year period (1988–2009), including the war period (1992–1995), and we retrospectively collected data on a total of 108 316 liveborn infants and their mothers from three different socioeconomic periods: before (1988–1991), during (1992–1995), and after the war (1996–2009). Association between incidence rate of low birth weight in liveborn infants and maternal sociodemographic status were determined for each study period. *Results*. There were 23 194 live births in the prewar, 18 302 during the war, and 66 820 in the postwar period. Among the liveborn infants born during the war, 1373 (7.5%) had birth weight of <2500 g, which is significantly more in comparison with 851 (3.6%) liveborn infants in this birth weight group born before and 1864 (2.8%) after the war. We found the number of examinations during pregnancy was 1.8 per pregnant woman in the war period, which was low in comparison with the number of examinations before (4.6 per pregnant woman) and after (7.1 per pregnant woman) the war (*P<.001* for both). Prewar perinatal mortality LBW infants of 6.2 per 1000 live births increased to 10.8 per 1000 live births during the war (*P<.001*), but after the war, perinatal mortality LBW infants (5.2‰) and early neonatal mortality (2.4‰) decreased. *Conclusions*. We found statistically significant association between low-birth-weight and maternal sociodemographic status in Tuzla Canton during 1992–1995 war in Bosnia and Herzegovina.

## 1. Introduction

Birth weight, like growth, is determined by the complex interplay of genetic and environmental factors. The proportional contribution of these influences is unclear. However, birth weight varies within genetically similar populations [1, 2], suggesting that environmental factors play a significant role. Low birth weight (LBW) is defined as weighing less than 2500 g (5.5 pounds) at birth, and is an important predictor of mortality and morbidity in the neonatal period [3], early postnatal growth [4], and growth during childhood [5]. It also has negative effects on cognitive and behavioral development in the first years of life, health status during childhood, and adult health [6]. Infants who are born with low birth weight (LBW; birth weight <2500 g) are divided into 2 categories: those who were born too early and those born too small. The 2 categories of LBW—preterm delivery (PTD) and intrauterine growth retardation (IUGR) separate infant health outcomes and have different causes [7]. Causes and risk factors for LBW, attributable to both PTD and IUGR, have been studied extensively, although earlier literature primarily grouped PTD and IUGR into the larger LBW category. In developing countries, most cases of LBW are attributed to intrauterine growth retardation (IUGR) rather than to preterm delivery. 

Maternal risk factors for having an infant with LBW in general include young age, unmarried marital status, less education, lower income, smoking, poor nutrition, and having had a previous infant with LBW [8, 9]. The association of other factors such as stress and exposure to some types of work during pregnancy remains unproven [10]. Other risk factors for LBW such as maternal age, although not themselves environmental factors, are strongly influenced by the social environment [11]. 

This has led researchers to search for new causes and risk factors for LBW. Recent literature has demonstrated that maternal stress, isolation, depression, and anxiety were associated with LBW [10–14]. Some studies have linked high maternal self-esteem, optimistic personality traits, and strong social networks with higher birth weight [15, 16]. Other studies, however, did not demonstrate association between birth weight and psychosocial factors after controlling for confounding variables, such as smoking [17, 18]. 

As much as 16% of all live births worldwide are LBW with a range of 3, 3–38%, >90% being in low-income countries. 

Birth weight figures are a useful parameter for assessing the effectiveness of prenatal medical care and indirect indicators of the share of at-risk newborns in the newborn population. Perinatal outcome is the measure of the quality of perinatal care given to the mother and child before, during, and after delivery. Low birth weight is a public health problem [19], and complicates around 17% of all births. It is the major risk factor for mortality in early infancy. The aim of this study was to examine association between incidence rate of low birth weight in live born infants and perinatal outcome with maternal sociodemographic status in Tuzla Canton during 1992–1995 war in Bosnia and Herzegovina.

## 2. Materials and Methods

### 2.1. Regional Characteristics

**Figure 1:**
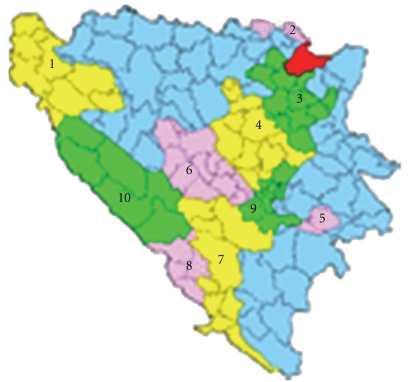
Map of Bosnia and Herzegovina; No 4-Tuzla Canton.

Population of 4.39 million of which 2.78 million lived on the territory of the present Federation of Bosnia and Herzegovina, one of the two political entities in the Republic of Bosnia and Herzegovina. Tuzla Canton (2909 km) has 13 municipalities and 510 353 inhabitants (Figure 1). 

Before the 1992–1995 war in Bosnia and Herzegovina, primary health care was provided in health centers and their outpatient facilities, secondary health care in general and regional hospitals and only partially in health care centers (specialized counseling), while tertiary health services were provided in medical centers which were also university teaching hospitals [20]. 

Perinatal care in Tuzla Canton had not been provided at the primary health care level mostly due to insufficient perinatal knowledge and clinical skills of primary care physicians and other staff, and a lack of adequate equipment and space as well. Thus, as an addition to tertiary health care, secondary health care which was easily accessible provided most of health care services to pregnant women, women giving birth, and newborns. All deficiencies of the health care system organization in Tuzla Canton became obvious during the war period. Regular examinations during pregnancy could not be performed at any level of health care, health care at birth was inadequate, and neonatal health care was almost nonexistent. Many hospital systems and the existing equipment were damaged or destroyed in war [21], shortages of medicines was evident, and a large proportion of health care staff either left the country or was needed on the battlefield. The existing hospital facilities were overcrowded with the wounded and patients with chronic diseases. The whole health care system was adapted to war circumstances. Perinatal care in Tuzla Canton rapidly deteriorated. Furthermore, there was a massive migration of the population due to the war and some parts of the country, such as Tuzla Canton, were overburdened with a large number of refugees and displaced persons.

### 2.2. Data Collection

The present study covers a 22-year period (1988–2009), including the war period (1992–1995), and it is based on the basis of the existing delivery protocols and medical histories of pregnant women treated at the Gynecology-Obstetrics Clinic in Tuzla. For measuring socioeconomic conditions of mothers we used definition that socioeconomic status (SES) is an economic and sociological combined total measure of a person's work experience and of an individual's or family's economic and social position relative to others, based on income, education, and occupation. When analyzing a family's SES, the household income earners' education and occupation are examined, as well as combined income, versus with an individual, when their own attributes are assessed [22]. Socioeconomic status is typically broken into three categories, high SES, middle SES, and low SES to describe the three areas a family or an individual may fall into. When placing a family or individual into one of these categories any or all of the three variables (income, education, and occupation) can be assessed. We retrospectively collected data on a total of 108 316 liveborn infants and their mothers from three different socioeconomics periods: before (1988–1991, middle SES), during (1992–1995, low SES), and after the war (1996–2009, low to middle SES). The available data with the known birth weights comprised 23 194 live births over the 4 years of the prewar period (5 798 annually), 18 302 live births over the 4 years of the war period (4 575 annually), and 66 820 live births over the 14 years of the postwar period (4 722 annually). We analyzed collected data such as the number of LBW, stillbirths, early neonatal deaths, perinatal deaths, gestational age, birth weights, and body length. 

We restricted the analysis to singleton liveborn infants of mothers who were between 15 to 49 years old, had complete information on birth weight, perinatal care, and age. Data about material and economic situation were not available during the war, because the refugees were allocated in collective accommodation and they together with domestic people had limited quantities of food. Data about marital status were not valid because pregnant women are traditionally married in Bosnia and Herzegovina, but during the war a lot of families were separated women had been raped and husbands were killed.

### 2.3. Perinatal Criteria

Intrauterine growth was estimated on the basis of birth weight and gestational age. Newborns were defined as low birth weight if their estimated birth weight was <2500 g at birth, irrespective of gestational age. Infants who are born with low birth weight (LBW; birth weight <2500 g) are divided into 2 categories: those born too early and those born too small. Standard practice in this institution is to weigh infants without diapers and with the umbilical cord shorter than 10 cm using spring scales to the nearest 100 g. We investigated the proportion of liveborn infants with low birth weight (<2500 g) by comparing the proportions of birth weight subgroups of 500–999 g, 1000–1499 g, 1500–1999 g, and 2000–2499 g in the pre-war, war, and postwar periods. Body length (cm) was measured from crown to heal in a newborn with completely extended legs and feet at the angle of 90°. The ponderal index was calculated as 100 × (birth weight (g)/birth length (cm)). Gestational age in weeks was estimated from the number of days between the first day of the last menstrual period and date of birth and from ultrasound examination if performed. If these data were not available, gestational age was assessed on the basis of external physical characteristics of the newborn [23]. Preterm birth was defined as a birth before completed 37 weeks of gestation. A postterm birth was defined as birth after completed 42 weeks of gestation. Stillbirth rate was defined as the number of stillbirths after 24 weeks of gestation per 1000 total births in the same time period. Ante partum stillbirths were those that occurred before the onset of labor, whereas intrapartum stillbirths were those that occurred during labor. The fetus was assumed to be alive at the beginning of labor unless there was evidence for otherwise. Early neonatal mortality was defined as the number of liveborn babies who died within the first 168 hours (7 days) after birth per 1000 live births. Perinatal mortality was defined as the number of deaths of newborns born after ≥24 weeks of gestation or with body weight of ≥500 g per 1000 live births [24].

### 2.4. Statistical Analysis

Differences in categorical data between the study periods were assessed by the *χ* ^2^ test. For 2 by 2 tables, rate ratios with 95% confidence intervals (CI) were calculated. The linear trend was calculated for the 500-g birth weight subgroup rates per 1000 liveborn infants with extended Mantel-Haenszel *χ* ^2^ test for trends. STATISTICA 6.0 (Stat Soft Inc., Tulsa, OK, USA) was used for all statistical analyses. *P* < .05 was considered statistically significant.

## 3. Results

Between 1988 and 2009, records on 108 316 singleton births were registered via medical childbirth notification forms. A total of 4088 (3.8%) mothers had infants who had LBW. During the war, 1373 (7.5%) mothers gave a birth to children with low birth weight (had birth weight of <2500 g) which is significantly more in comparison with 851 (3.6%) mothers who gave a birth to children with LBW before (rate ratio 2.129; 95% confidence interval (CI); 1.950–2.324) and 1864 (2,8%) after the war (rate ratio 2.826; 95% confidence interval (CI); 2.630–3.036). During the war, in the group of term infant 4.5% of liveborn had LBW, which is significantly more in comparison with the two other groups before 1.8% and after the war 1.1% (*P* < .0001). However, during the war, more infants had LBW because the IUGR, while in the two other period more infants had LBW because of the preterm delivery (PTD). During the war of 39.5% of mothers with LBW had preterm labor (<37 weeks) and in comparison to the other two periods (45.5% and 47.4%) the difference is statistically important (*P* < .0001 for both periods). Bigger number of mothers who gave birth with LBW on time (51.1%) and after term (9.1%), which is in comparison to period after and before war important statistic difference (Table 1). However, 92 mothers gave a birth to stillborns with LBW during the war and in comparison to mothers before the war, prevalence among stillborns with LBW was on the edge of statistical importance while in after war period prevalence of stillborns with LBW was smaller. There is significant difference before and during the war (*P* = .04), but it significantly decreased after the war (*P* = .0001). 

The proportion of deliveries attended by health care providers was 75.9% during the war, which was significantly lower than before (99.1%) and after (99.5%) the war (*P* < .001).

The number of examinations during pregnancy was 1.8 in the war period, which was low in comparison with the number of examinations before (4.6) and after (7.1 per pregnant woman) the war (*P* < .001 for both).

We found that 7.9% of mothers with LBW had first-trimester prenatal care during the war compared with 52.4% of mothers with LBW before (rate ratio: 0.078; 95% confidence interval (CI): 0.061–0.099) and 33.8% after the war which was statistically significant (rate ratio: 0.168; 95% confidence interval (CI): 0.135–0.209, resp., (Table 2)). During the war, 68.9% mothers who delivered infants with LBW began PNC in third trimester, which is a statistically significant difference comparing to mothers who delivered infants with LBW before (Rate ratio 11,193; 95% confidence interval (CI): 0.038–13.862) and after the war; (rate ratio 5.854; 95% confidence interval (CI): 5.023–6.822). There was statistically significant association between LBW and number of examinations during pregnancy. 

The early neonatal deaths, 5.8 per 1000 total births was significantly higher during the war than before the war 2.1 per 1000 total (*P* < .0001), but after the war 2.4 per 1000 total births they did not have significant difference (*P* < .448). Perinatal mortality rate of 10.8 per 1000 total births was significantly higher during the war than before and after the war (6.2 and 5.2 per 1000 total births, respectively; *P* < .05 for both). 

The proportion of liveborn infants with birth weight of 500–999 g during the war did not have significant difference in comparison to the other two periods (*P* < .239). The proportion of liveborn infants with birth weight 2000–2499 g (50.9%) was the highest after the war. The average birth weight, birth length, and gestation age of newborns with LBW during the war was significantly lower than before and after the war (Table 3). Average weight of newborns with LBW during the war in relation to gestation age and Ponderal index suggests that LBW occurred not because of preterm birth, even though average gestation age was less than 37 weeks, but for unsatisfactory intrauterine growth or stagnation in intrauterine growth. Ponderal index also suggests the second one.

Mothers with infants who had LBW were during the war more likely to be Muslim, refugees, had less than a high school education, married, and mostly younger than 20 years. The number of mothers between 25–29 years with infants who had LBW was noted during the war was by 5% lower than their number before the war, but not very different from the number after the war (Table 2). Significantly higher number of mothers aged 35 and older (28.9%) was during the war than before and after the war (18.6% and 20.5%, resp., *P* < .0001 for both).

## 4. Discussion

Our results suggest that the impact of socioeconomic status on birth weight is mediated through the differential utilization of prenatal care and access to medical facilities. We found that the number of newborns with LBW in Tuzla Canton significantly increased during the 1992–1995 war. During the war, Bosnia and Herzegovina did not introduce any measures (e.g., increase the level of health consciousness in collective centres and distribute larger quantities of humanitarian aid to pregnant women) to counteract the unfavourable socio-economic situation at least partly. 

We believe that a low maternal sense of control was associated with LBW which occurrence increased to 7.5% compared with the prewar rate of 3.6% and 2,8% after the war. However, during the war more infants had LBW because of the IUGR, while in the two other periods more infants had LBW because of the preterm delivery (PTD). Low birth weight and preterm delivery are the most important determinants of neonatal mortality, as well as infant and childhood morbidity. LBW contributes to approximately 65% to 75% of neonatal deaths [25]. We found perinatal mortality rate LBW of 10.8 per 1000 liveborn infants was significantly the highest during the war, as well as early neonatal mortality rate. However, the rate of stillborns was significantly higher during the war than before and after the war. 

The incidence of low birth weight shows a marked geographical pattern [26, 27]. More than 20 million infants worldwide, representing 15.5 per cent of all births, are born with low birth weight, 95.6% of them in developing countries. The level of low birth weight in developing countries (16.5%) is more than double the level in developed regions (7%). Half of all low birth weight babies are born in South Central Asia, where more than a quarter (27%) of all infants weighs less than 2,500 g at birth. Low birth weight levels in sub-Saharan Africa are around 15 per cent. Central and South America have, on average, much lower rates (10%), while in the Caribbean the level (14%) is almost as high as in sub-Saharan Africa. About 10% of births in Oceania are low birth weight births [28–31]. A geographical pattern characterized the incidence of low birth weight in Europe, with lower rates in the more northerly countries. 

The relationship between maternal antenatal education and perinatal outcomes is well established. Our study shows that during the war, 68.9% of mothers who delivered infants with LBW began PNC in the third trimester, which is statistically significant difference in comparison to mothers before 16.6% and after the war 27.6%. If we add other negative factors related to war condition it seems that insufficient health care for pregnant women and newborns in Bosnia and Herzegovina in war circumstances, combined with poor socioeconomic conditions, constant fear, stress, and destruction of homes and families, was the main factor accounting for the increase in number of mothers who gave a birth to a children with LBW during that period.

The beneficial effects of prenatal care have been documented in many observational studies over several decades [32–35]. Studies that have assessed the influence of advanced maternal age on LBW, preterm, and IUGR deliveries among primigravidas have provided conflicting and inconclusive results [36]. In our study, as a result of the increasing proportion of births in women aged 35 years and older during the 1992–1995 war perhaps influenced to increase in number of children with LBW. So the main factors of LBW were fewer examinations of women during pregnancy, more unattended deliveries, and perinatal care in country during war period.

Rohrer's ponderal index in newborns was used as an indicator of fetal growth status and lower ponderal index, as in our study, indicates a strong impact of maternal factors with LBW [37]. 

Several limitations should be considered when assessing our results. There is limited earlier literature on the role of sense of control and pregnancy outcomes, especially IUGR. 

The limitations of our study were incomplete data on the parity and pathological conditions of mothers and children during pregnancy, such as pathology associated with premature childbirths and intrauterine growth retardation with consequential low birth weight and hypertrophy of infants. We were also unable to examine risk factors such as chronic and comorbid conditions, congenital malformations, obstetric complications, and infections. 

Limited accessibility and quality of perinatal health care during the war had detrimental effects on infants' weight. Associations between other psychosocial factors and IUGR should be explored further for better understanding of how stress and psychosocial resources have an impact on fetal growth and possibly in a prospective manner to limit potential biases.

## 5. Conclusions

Our results suggest that the impact of socioeconomic status on birth weight is mediated through the differential utilization of prenatal care and access to medical facilities. During the war, Bosnia and Herzegovina did not introduce any measures (e.g., increase the level of health consciousness in collective centers and distribute larger quantities of humanitarian aid to pregnant women) to counteract the unfavorable socio-economic situation at least partly. Higher number of low birth weight newborns during the war had influence on current surveillance, but long-term effects will be seen in the future. Further investigations in Tuzla Canton should be in that direction with the aim of showing the ventual consequence on population over these years.

## Figures and Tables

**Table 1:** Number of Low birth weight (LBW) infants according to gestational age, stillbirths, early neonatal mortality, and total perinatal mortality Low birth weight (LBW) infants at University Clinical Center Department for Gynecology and Obstetrics in the prewar (1988–1991), war (1992–1995), and postwar (1996–2009) period.

Group of Newborns with LBW	No. of newborns with LBW				
	Prewar (*n* = 851/23194)	P*	War (*n* = 1373/18302)	P*	Postwar (*n* = 1864/66820)
Gestational age (weeks):			
<37	387 (15, 0%)	.0001	542 (23, 8%)	.0001	883 (13, 1%)
37–42	375 (1, 8%)	.0001	705 (4, 5%)	.0001	670 (1, 1%)
>42	89 (16, 7%)	.0001	126 (31, 8%)	.0015	311 (23, 9%)
Stillborns^†^	96 (5.1‰)	.04	92 (5.0‰)	.0001	187 (2.8‰)
Early neonatal deaths^†^	49 (2.1‰)	.0001	107 (5.8‰)	.448	165 (2.4‰)
Perinatal death^†^	145 (6.2‰)	.0001	199 (10.8‰)	.043	352 (5.2‰)
*PI	**1.8**		**1.7**		**1.7**

*χ ^2^1 test.

^†^The number in the parentheses is the number of stillborns per 1000 total births.

*PI ponderal index (PI = weight (in g)/length (in cm)^3^).

**Table 2:** Maternal age and prenatal care of follow up and birth data for Low birth weight at University Clinical Center of Tuzla, Department for Gynecology and Obstetrics in the prewar (1988–1991), war (1992–1995), and postwar (1996–2009) period.

Maternal characteristics-Maternal age (years)	Proportion (%)			Rate ratios (95% confidence interval)			
	Prewar (*n* = 851)	War (*n* = 1373)	Postwar (*n* = 1864)	Prewar	*P*	Postwar	*P*
<20	163 (19.1%)	275 (20%)	326 (17.5%)	1.057 (0.851–1.311)	.614	1.181 (0.988–1.412)	.06
20–24	259 (30.4%)	332 (24.2%)	660 (35.4%)	0.72 (0.602–0.882)	.001	0.679 (0.581–0.497)	.0001
25–29	271 (31.8%)	369 (26.8%)	496 (26.6%)	0.78 (0.652–0.948)	.01	1.013 (0.866–1.186)	.86
≥30	158 (18.6%)	397 (28.9%)	382 (20.5%)	1.78 (1.448–2.197)	.0001	1.578 (1.341–1.855)	.0001
Trimeste prenatal care							
First trimester prenatal care	446 (52.4%)	109 (7.9%)	631 (33.8%)	0.07 (0.061–0.099)	.0001	0.168 (0.135–0.209)	.0001
Second prenatal care	264 (31%)	317 (23.2%)	720 (38.6%)	0.667 (0.550–0.808)	.0001	0.476 (0.407–0.557)	.0001
Third or No Prenatal care	141 (16.6%)	947 (68.9%)	513 (27.6%)	11,193 (0.038–13.862)	.0001	5.854 (5.023–6.822)	.0001

Rate ratios for an association between maternal age during pregnancy and low birth weight (LBW). CI = confidence interval. a Chi-squared test for linear trend used for comparison of proportions and linear-contrast analysis of variance used for comparison of means.

**Table 3:** Average birth weight, body length, gestation age, and Ponderal index of LBW newborn in the Clinic for Gynaecology and Obstetrics during the prewar (1988–1991), war (1992–1995) and postwar periods (1996–2009).

LBW Average	Proportion (%)			Prewar	Postwar
	Prewar (*n* = 23194)	War (*n* = 18302)	Postwar (*n* = 66820)	P**	P**
Birth weight	2063.49	1857.52	2015.85	.0003	.0001
Body Length	48.4	47.5	49.2	.0001	.0001
Gestation age	35.9	36.5	37.6	.0001	.0001

**Independent samples *t* test.
